# Stem cells and anti-aging genes: double-edged sword—do the same job of life extension

**DOI:** 10.1186/s13287-017-0746-4

**Published:** 2018-01-10

**Authors:** Mujib Ullah, Zhongjie Sun

**Affiliations:** Department of Physiology, BMSB, College of Medicine, University of Oklahoma Health Sciences Center (OUHSC), 940 Stanton L. Young Blvd, Oklahoma City, OK 73126-0901 USA

**Keywords:** Lifespan, Rejuvenation, Klotho, Stem cells, Aging communication, Cytokines, Genetics, Telomerase, Aging signaling, Caloric restriction

## Abstract

Aging impacts diseases and lifespan. With current knowledge of stem cells, it is feasible to design and test interventions that delay aging and improve both health and lifespan. Stem cells, together with anti-aging genes such as Klotho, play a crucial role in delaying the aging process. Stem cells in combination with anti-aging genes make a complex and protective shield, which stand against the eroding effects of aging. Increased wear and tear of the stem cells, as well as Klotho deficiency, is expected to heavily increase cellular damage and accelerate the process of aging. Stem cells in conjugation with anti-aging genes probably receive and neutralize most of the devastating signaling effects which are known to cause premature aging. The shield of stem cells combined with anti-aging genes is a primary target for absorbing the shock of aging. If this shield neutralizes the shocks, it could lead to a youthful state, but if not it will accelerate the aging journey. In this review, we concisely discuss the neutralizing ability, operated and regulated by stem cells and other life-extension factors. We suggest that stem cell interventions that increase rejuvenation and keep in balance the expression of anti-aging genes could delay the aging phenotypes and result in prolonged lifespan.

## Background

Aging is an unavoidable but extendable process [1]. Aging brings several diseases together for devastating effects on human society and overloads the entire health care system and economy [1, 2]. Arthritis, cardiovascular disease, cancer, dementia, osteoporosis, diabetes, hypertension, degeneration of tissues, neuropathy, stroke, obesity, and depression are examples of age-induced diseases [1, 2]. Aging is a vital trigger of diseases and extends their shocks to affect vision, hearing, muscular strength, bone mass, immunity, nerve function, and metabolic disorders [3]. Aging is directly a face-to-face threat to life itself and also poses enormous challenges to the entire system, thereby necessitating an urgent need to address these health concerns.

## Aging and stem cells

**Fig. 1:**
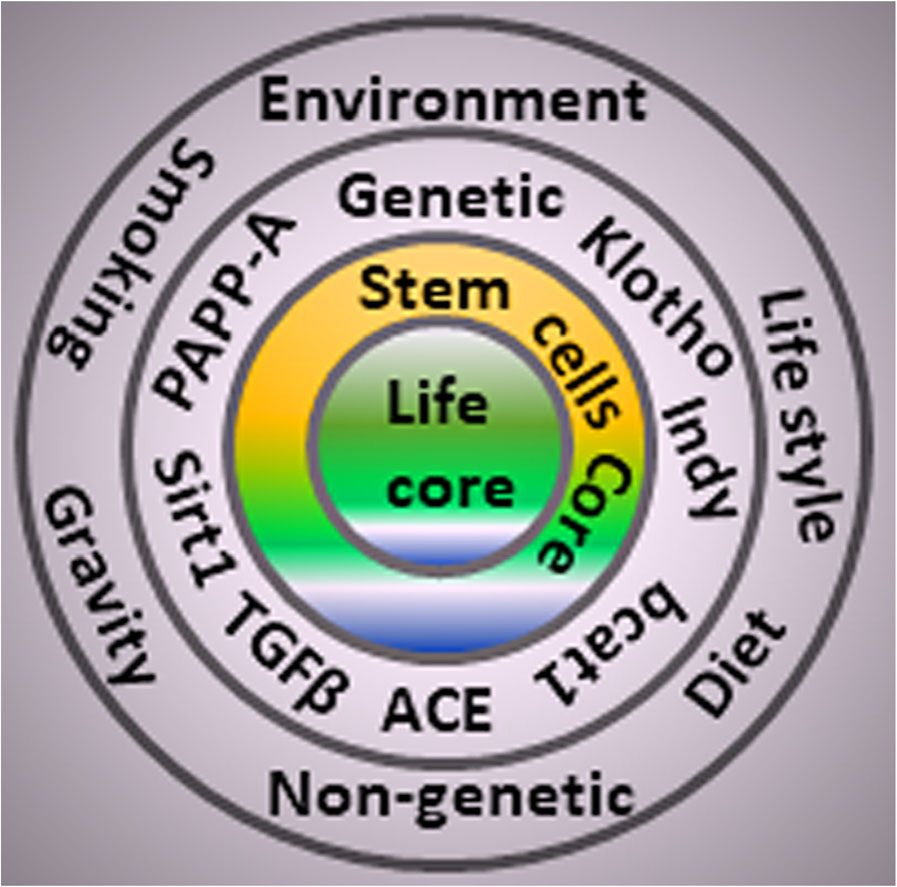
Factors affecting the core of life. Different circles represent the different categories of factor responsible for affecting aging and longevity. Outside circle consists of environmental, lifestyle, gravity, chemicals, drugs, geographical regions, and diet-related factors. Second circle is representative of genetic and vital genes such as Klotho, and I am not dead (Indy). The third core shows rejuvenating stem cells are very close for renovation of damaged cells for the core of life. All circles eventually accumulate the burden of damages, mutations, diseases, and degeneration on the central core of life (aging). ACE angiotensin-converting enzyme, PAPP-A pregnancy-associated plasma protein-A, TGFβ transforming growth factor beta and Sirtuin 1 (Sirt1)

**Fig. 2:**
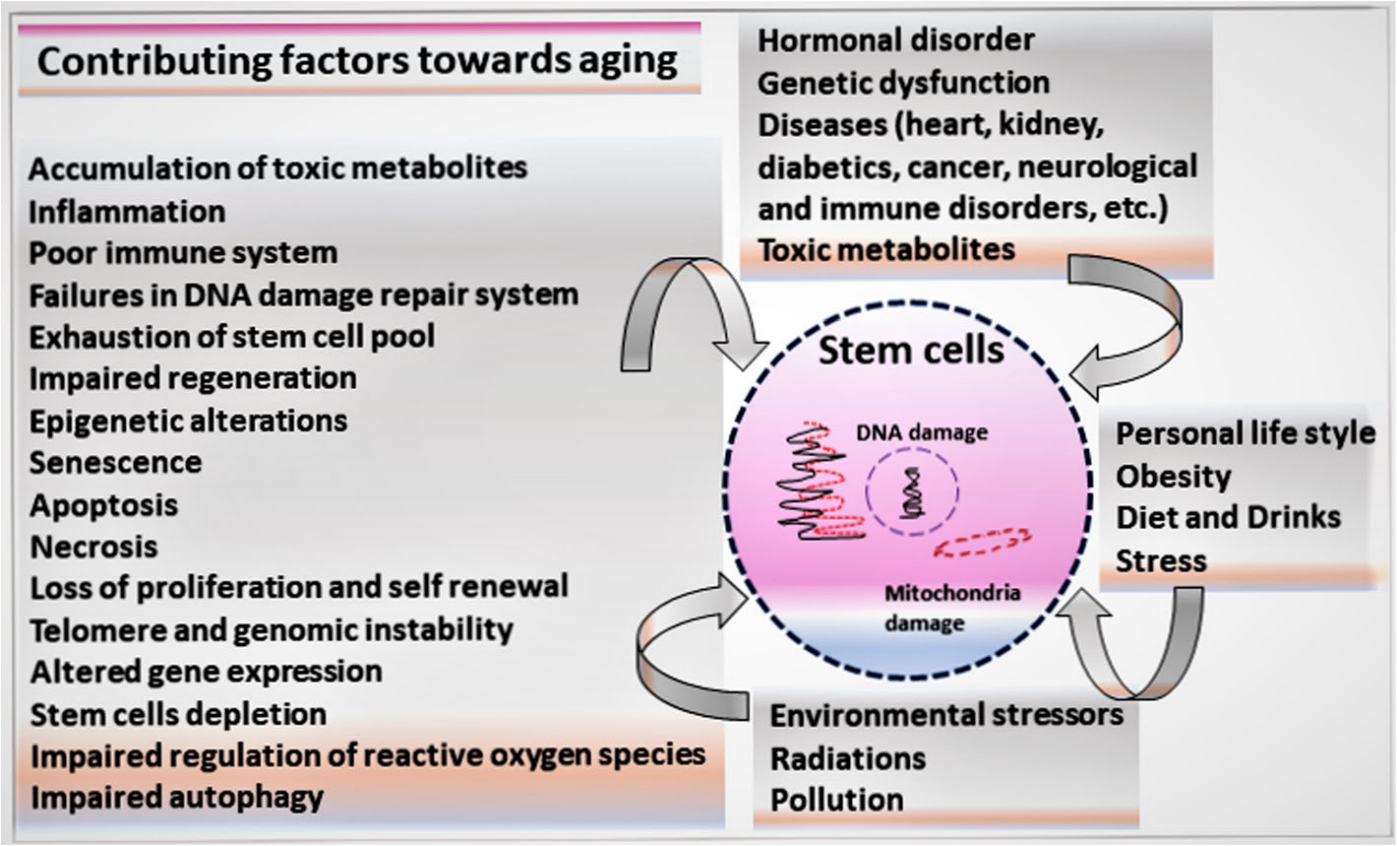
Factors contributing to aging. Factors influencing the aging process: environmental stressors, diet, genetic, nongenetic, hormonal, and epigenetic

Aging is a complex process, which deteriorates body functions such as loss of skin elasticity, accumulation of fats and atherosclerosis, decline of the immune system, bones prone to fractures, and eventually death [1, 3]. Aging is determined by a complex mixture of genetic, nongenetic, and environmental factors [1, 2]. Eventually, all of these factors whether known or unknown accumulate their message of aging in the core of stem cells coordinated by a second core of life-extending genes (Figs. 1 and 2). Hence, all aging phenomena could be interpreted on the basis of stem cells [1, 2]. They are the regenerative building blocks and seeds of life [4, 5]. They enhance the restorative prowess of living organisms. Stem cells could be a backup system for the living organism to replace damaged tissue or worn-out cells [1–3]. Thus, stem cells could be a core factor in deciding aging. In the animal kingdom, humans have reached the top positions but have lost the mainstream regenerative power found in lower animals [6]. Have stem cells given up their regenerative ability in the journey of evolution from protozoa to mammals [2, 6]? For instance, lower animals have an amazing regenerative power. The speed of this regeneration is 5 days in Planaria [7] and 7–10 days for complete new body formation in Hydra [8]. Salamanders recovered their lost limbs within days [9]. All of these animals have a big pool of stem cells or have otherwise reprogrammed their differentiated cells into stem-like cells [9]. According to one estimation, stem cells comprise about 20% of flat worm body cells, and a Hydra looks like a permanent embryo [10]. Salamanders and other vertebrates use the reprogramming and dedifferentiation machinery for regeneration of their missing/injured parts [9, 10].

Mammals, especially humans, partially rejuvenate their diseased or missing parts by using stem cells. Similarly, hematopoietic stem cells continuously maintain their population to renew blood cells [11]. The use of stem cells in aging aims to understand their mechanisms and how the regenerative capacity of stem cells can be enhanced to promote healthy life [11, 12]. According to one theory, stem cells from the bone marrow go into tissue and become cells of that tissue [13]. Aging is a balance between entropy (aging or slow breaking down of tissue) and rebuilding (stem cells). If the power of entropy is bigger than the rebuilding abilities of stem cells, this will lead to aging [13]. Stem cells replace damaged cells every day, but the magnitude of damage in aging is bigger than the capacity of stem cells [12]. How stem cells and anti-aging genes take part in the process of life extension requires further investigation. Here, we describe these aging factors, the ways in which they interact, and how these interactions decrease stem cell health over time. A better understanding of these changes will lead to unfolding aging mysteries (Figs. 1 and 2).

## Aging and anti-aging factor Klotho

Stem cells are not the only substances that entirely control the core of life; other compounds, especially genetic factors, also play a substantial role to keep the fountain of life constant with aging [14]. Klotho is one of the most well-studied genes for extension of life in many animal models [14]. In Greek mythology, three goddesses named Klotho, Lachesis, and Atropos determine, decide, and control the thread of lifespan [15]. Among them, the goddess Klotho spins and controls the thread of life [15]. Klotho has been identified as an age-suppressing gene, and mice enjoyed an extended lifespan when it was overexpressed [14, 16]. Premature aging was observed when Klotho expression was disrupted or repressed [3, 17]. Several functional aspects of the Klotho gene have been investigated in the context of direct aging and indirect aging (playing a vital role in multiple life-oriented diseases) [17]. Klotho overexpression heavily influences the process of proliferation, oxidation, senescence, autophagy, and modulation of insulin, TGF-β, and Wnt signaling pathways [14, 15, 17]. Klotho deficiency activates Wnt expression and contributes to the senescence and depletion of stem cells, which consequently triggers tissue atrophy and fibrosis [18, 19]. A better understanding of the potential effects of Klotho on stem cells could offer novel insights into the cellular and molecular mechanisms of aging and diseases. As has been reviewed robustly, Klotho overexpression extends the lifespan and defective Klotho results in rapid aging and early death in mice [18, 20]. Klotho deficiency accelerates cardiovascular damage, impairs endothelial function, fades vasodilation, dwindles angiogenesis, changes cellular calcium hemostasis, and disturbs the bone-matrix structure that eventually results in premature aging [3, 16].

## Aging and contributing factors

Aging and longevity are determined by a complex combination of genetic, nongenetic, and environmental factors [21]. On the genetic side, Klotho and other genes have been demonstrated useful for life extension and in animal models, where single-gene mutations linked with insufficient expression have been shown to dramatically decrease the lifespan in mice [2, 20, 22, 23]. These genes are useful potential targets for interventions of aging, which could probably do the same job in human longevity.

From the environmental perspective, diet (specifically, caloric restriction) has been shown to substantially affect lifespan, including delay or prevention of many aging-related diseases [21, 24]. Dietary restriction promotes longevity in many species, and delays aging at the genetic level by stabilizing telomerase activity [21, 25]. The effects of caloric restriction counteract the effects of oxidation, inflammation, detoxification, telomerase activity, and decreasing stress and damage to genes [12, 25, 26]. Mice that were fed a lower calorie intake showed a reduction in the incidence of age-related diseases [21, 25]. Further investigation of the diet and caloric restriction on aging and telomerase could help unveil new anti-aging treatments.

Among many factors that affect the aging process, environmental exposure is one of the major modifiable risk factors [24]. Environmental exposures, pollutants, and chemicals together influence aging, frequently resulting in development of certain diseases at an earlier age than expected. This is a long list, not only limited to various toxins, pollutants, gravity, and climate changes, but extended to personal lifestyle as well in the form of smoking, drinking, stress, social burden, and depression [21, 24]. Genetic and environmental factors aggravate the process of aging, possibly due to accumulated damage over a lifetime [27]. However, the regenerative power of stem cells limits the decay process of aging [11] but stem cell depletion occurs with increasing age [2, 12]. Moreover, when stem cells divide, their telomeres shorten, and cells stop dividing and die [2, 28]. Telomerase prevents this decline in stem cells, by lengthening telomeres, but the diminished activity of this enzyme has been reported in the aging process [28, 29]. When mice are engineered to lack telomerase completely, their telomeres progressively shorten, and the animals age much faster than normal mice [25, 28].

Telomere shortening has been reported to cause severe impairment of stem cell mobilization and higher genomic instability, and is correlated with decreased longevity in various organisms [12, 28]. Mice deficient for telomerase activity or telomere regulating proteins are characterized by accelerated aging phenotypes and stem cell dysfunction [28]. Moreover, conditions that trigger premature aging, such as telomere shortening, also impair the ability of stem cells to regenerate tissues. Short telomeres increase the likelihood of cells becoming senescent and producing molecules that lead to inflammation, which is a huge risk factor for age-related diseases [25, 28]. Stem cells, owing to many cellular divisions, have the ability to activate telomerase and regulate the length of the telomeres [28, 29]. Because telomerase works for tissue healing, there is hope in the future that telomerase expression could be used for intervention approaches for certain telomere diseases or aging syndromes. But all aging syndromes are not due to telomeres; other factors of health span such as food, sleep, exercise, caloric restriction, lean body mass, stress, and impaired function of stem cells will also accelerate the aging process. Stem cells from different tissues have a high grade of variability to heal and regenerate [30]. For instance, the nervous system has weak powers of regeneration, while the skin has high power and muscle cells have intermediate ability of regeneration. Heart tissue and their stem cells have feeble regenerative capabilities [31–33]. However, recent discoveries about stem cells are beneficial to understand the restorative abilities of tissues.

## Aging and signaling pathways

Aging is regulated by a complex series of specific signaling cascades. For example, stem cells use insulin-like growth factor 1 (IGF1) for repair and regeneration. Overexpression or elevated activity of such a growth factor is required to improve muscle regeneration [34]. The signaling events of growth hormone are required to control the proliferation and development of tissues. Klotho has been shown to act as a circulating hormone and repress the insulin (IFG1) pathway for extension of life in mice [20]. Downregulation and mutations in the TGF-β signaling pathway or in the TGF-β receptors (TGF-βR1/R2) extended worm lifespan by about 20% [35]. Hormonal signaling pathways are extremely potent regulators of lifespan, and they coordinate the longevity of several key organs by acting in a systemic manner along with other pathways. These include steroid signaling, sirtuin deacetylases, nutrient sensing, AMP-activated protein kinase, ROS signaling, tumor suppressor, and stress-induced protein kinases [1, 35, 36]. Anti-aging treatments are required to increase the number and quality of stem cells and also to activate the regenerative signals in them [1, 35, 36]. Regenerative stem cells or anti-aging supplemented treatments potentially slow down or reverse this process by improving immune rejuvenation and repairing functions [12]. Stem cells after injections at injury sites improved the restorative ability of tissue repair [12].

Different stem cells have diverse regenerative abilities. As an example, hematopoietic stem cells have the highest grade repairing feature to continuously renew cells in the main blood stream. Similarly, embryonic, pluripotent, and mesenchymal stem cells (MSC) are related to tissue repair [3]. It is hypothesized that stem cells secrete some kind of perplexing anti-aging substances. If we can identify these, we have found an anti-aging protein for life extension.

## Aging and genetics

Many genes are involved in the determination of destiny of lifespan. What are these genes, and how do they take part in the underlying mechanism of life delay? About 1% of genes could participate in the aging and longevity process of *Caenorhabditis elegans* [37]. The *bcat-1* gene is one of them. On blocking its effect, the lifespan of the *C. elegans* worm was extended by about 25% [27, 35, 37]. In higher animal models, researchers have dramatically extended the lifespan by modulating the expression of a few genes. Klotho is an example of such a gene. Other genes involved in the promotion of growth, maturation, and fertility include *Prop-1*, *Pit-1*, and GH-R/GHBP, which have a role in determining the onset of aging; when these genes are silenced, aging is delayed [27, 38]. Limited expression of angiotensin-converting enzyme (ACE), adenylyl cyclase type 5 (AC5), angiotensin II receptor type 1 (Agtr1a), apolipoprotein A-1, I am not dead yet (INDY), and pregnancy-associated plasma protein-A (PAPP-A) is associated with longer life. Similarly, the overexpression of AMPK, activating transcription factor 4 (ATF4), cyclin A2, FGF21, follistatin, FOXN1, GDF11, interleukin-21 (IL-21), Klotho, Oct4, PGC-1, SUMO-1, TGF-β1, and uncoupling proteins (UCP) has a direct or indirect role in the long life destiny [27, 38–40]. Genes and their encoded proteins play a substantial role in the longevity of life by altering their secretion of important growth factors, cytokines, and enzymes [41]. In fact, the amount of secretion controls the cell destiny toward wear and tear [41]. This is a highly complicated and interdependent set of factors that is still unknown, and further studies are needed to understand the biology of life extension.

MSC target a specific tissue because they require the right combination of signaling molecules from the injured tissue and the corresponding receptors [12]. Using signaling tactics, MSC have been demonstrated to respond to various situations and even in some cases to reside in injured tissues [12].

How do MSC know when to respond, where to go, how to communicate, why to stay at destination and what to do there, and how to judge their fate whether needed or not? To better understand this, we need to unravel the complex network of stimulatory signals. These stimulatory signals could be from origins like cytokines or/and growth factors. In fact, stem cells use the communicative language of cytokines and growth factors during the regenerative process [41]. Like an emergency siren, injured tissues secrete cytokines and growth factors to attract stem cells and use them in such a way as to promote healing and regeneration [41]. For instance, protein-rich plasma helps stem cells to increase their population both in quantity and function. The secretion of FGF2 and VEGF (growth factors) is well documented for homing stem cells to injured areas of the body [26, 41]. They also stimulate blood vessel formation by attracting stem cells [42]. Bone morphogenetic protein has the ability to divert cellular traffic of stem cells toward bone formation. Fibroblast growth factor empowers the proliferative potential of stem cells to cope with an emergency demand during regeneration and repair [26, 42].

As first responders, stem cells migrate to the place of damage and coordinate the rescue operation [42]. Stem cells become the regenerative portion of residing tissue, but otherwise the healing effects are due to their secretory cytokines and growth factors in response to the injury. Additionally, stem cells communicate with local tissues and direct their reserve cells and mediators to coordinate and control the steps of damage and repair [41, 42]. However, the sword of aging cuts this amazing reparative ability of stem cells with time. As we can see, there is a remarkable power of regeneration at childhood, but the regenerative pace decreases with progressive aging [3]. Over the lifespan, the number of stem cells declines dramatically, and provides a reasonable explanation of why healing capacity reduces with aging.

## Language of aging

Stem cells and life-extending factors probably use the language of cytokines, growth factors, inflammatory signals, and other mediators for communication between the damaged tissue and the rescue team of stem cells and other healing cells [26]. This communication is not limited to one another and with other cells, but is also extended to neighbor cells and tissues, and even the entire body [41]. MSC are well known to modify their cytokine output based on what is needed, where it is needed, at which time it is needed, in which amount it is needed, how to neutralize inflammation, and how to regulate anti-inflammation in response to effective communication [26, 41]. However, this communication system weakens with increasing age and further accelerates the aging process. Effectively, understanding and targeting such communication lines may be a better choice for future aging therapies.

## Aging and cosmetics

Apart from diseases and longevity, aging biology will revolutionize the whole cosmetic industry by renewing and growing a new healthy skin [43, 44]. At any age, healthy skin is seen as younger and aesthetically more appealing than unhealthy skin. Again, we may not be younger, but we can look younger. With increasing age, the quantity and function of stem cells decline dramatically and is an explanation for why healing capacity diminishes over the lifespan [3, 43, 44]. However, in aging the number of MSC fades in the bone marrow, which is one of the major causes of aging [3, 44]. Alternatively, if more stem cells are present in the body, more are available to migrate to damaged areas and repair them by secretion of growth factors, which could be a powerful new class of active ingredients for skin therapeutics. They should be able to promote efficient and scar-free healing. Harvesting the growth factors would be useful in the fight against damaged skin. For scar removal, we may use the example of macrophages, which reach the target area and clean damage and debris [45].

## Conclusion

Aging is a worldwide disaster leaving nothing behind in a youthful state forever, but in the future it could probably be delayed for some time using the latest scientific discoveries. These discoveries would introduce the latest technologies to control and reprogram the genes and stem cells involved in aging. Restoration of youthful gene expression would enable cells to grow younger. Restoring the youthful gene expression may help turn back the clock on aging and potentially extend the healthy lifespan. Responsible factors could be of a genetic, nongenetic, and environmental origin [2, 27]. On the genetic side, this could involve modulating the expression of Klotho, AMPK, cyclin A2, FGF21, FOXN1, GDF11, Oct4, PGC-1, TGF-β1, apolipoprotein A-1, and I am not dead yet (INDY), the genes responsible for life extension [38–40].

On the environmental side, foods, nutrients, vitamins, and medications make us healthier probably because they induce the expression of health-promoting genes and reduce the expression of disease-promoting genes [21, 24, 46]. Conversely, the consumption of toxic substances including chemicals, smoke, drugs, and alcohol do the reverse.

Regenerative therapies concentrate their focus on stem cells and other life-extending factors and seek attention to reverse aging. Nevertheless, the potential of stem cells to be used as therapeutic cargo has had a profound effect on the vision of the future of regenerative medicine. Aged tissues have a limited stem cell reservoir, decreased population, and low renewal efficiency [11, 30]. All of these are the contributing factors for cellular senescence, apoptosis, autophagy, and oxidative stress, which finally accumulate and lead to aging [27, 47, 48].

Anti-aging therapies are a new emerging era of science that seem to benefit society using the power of stem cells, cytokines, and growth, regenerative, and life-extending factors. Future scientific discoveries will unravel these puzzles of aging biology. Probably, such life-extending factors mobilize the endogenous stem cells or renew or increase their number and functions [1]. Regenerative therapies and exogenous stem cell transplantation into damaged tissues will also improve the wear and tear of aging.

Lessons from cancer biology may be vital to quench the historical quest for immortality [29, 49]. It seems that both cancer and aging share antagonistic features, and inhibition of one can cause the activation of the other. Cellular senescence, apoptosis, telomere shortening, and other mechanisms that protect us from cancer might accelerate aging [49, 50]. Similarly, longevity requires indefinite cell proliferation which might trigger cancer [45]. Nonetheless, aging and cancer are tightly interconnected and seem to share common biological features. Now it is clear that both cancer and aged cells show DNA damage, genomic instability, and mutations [29, 49]. How do cancer cells fuel aging or how does aging fuel cancer? Are both cancer and aging the victims or the culprit of stem cell deregulation? Answers may push one more step forward in understanding the aging biology. Application of stem cell therapeutics to delay the aging process by improving cures for disease is clearer, but extension of human life apparently seems more remote. While animal models are well studied for extension of life [15], such translation from animal into human is more challenging.

Aging is not just due to local wear and tear. It is a process (a cumulative process of damage), and the process may be controlled in a significant way. Stem cells are an excellent candidate for regenerative medicine; however, it is important to understand that these miraculous cells may indeed be the future of medicine for mainstream cellular therapies of aging. Aging science is in its infancy, but it is clearly leading to the time when scientists will develop genetic engineering and stem cell therapies that will enable us to reverse aging and help us to grow younger and healthier.

## Abbreviations

AMPK: Adenosine monophosphate-activated protein kinase
FGF2: Fibroblast growth factor 2
FOXN1: Forkhead box N1
GDF1: Growth differentiation factor 1
IGF1: Insulin-like growth factor 1
INDY: I am not dead yet
KL: Klotho
MSC: Mesenchymal stromal cells
Oct 4: Octamer-binding transcription factor 4
PGC 1: Proliferator-activated receptor-gamma coactivator-1
ROS: Reactive oxygen species
SUMO1: Small ubiquitin-like modifier 1
TGF-β: Transforming growth factor beta
Wnt: Wingless-type MMTV integration site family member
